# Extended Field Laser Confocal Microscopy (EFLCM): Combining automated Gigapixel image capture with *in silico *virtual microscopy

**DOI:** 10.1186/1471-2342-8-13

**Published:** 2008-07-16

**Authors:** Emilie Flaberg, Per Sabelström, Christer Strandh, Laszlo Szekely

**Affiliations:** 1 Department of Microbiology, Tumor and Cell Biology (MTC) and Center for Integrative Recognition in the Immune System (IRIS), Karolinska Institute, Box 280 S-17177 Stockholm; 2 Sweden Karolinska Institute Visualization Core Facility (KIVIF); 3 Aragon System Poppelvägen 11 SE-832 54 Froson Sweden

## Abstract

**Background:**

Confocal laser scanning microscopy has revolutionized cell biology. However, the technique has major limitations in speed and sensitivity due to the fact that a single laser beam scans the sample, allowing only a few microseconds signal collection for each pixel. This limitation has been overcome by the introduction of parallel beam illumination techniques in combination with cold CCD camera based image capture.

**Methods:**

Using the combination of microlens enhanced Nipkow spinning disc confocal illumination together with fully automated image capture and large scale *in silico *image processing we have developed a system allowing the acquisition, presentation and analysis of maximum resolution confocal panorama images of several Gigapixel size. We call the method Extended Field Laser Confocal Microscopy (EFLCM).

**Results:**

We show using the EFLCM technique that it is possible to create a continuous confocal multi-colour mosaic from thousands of individually captured images. EFLCM can digitize and analyze histological slides, sections of entire rodent organ and full size embryos. It can also record hundreds of thousands cultured cells at multiple wavelength in single event or time-lapse fashion on fixed slides, in live cell imaging chambers or microtiter plates.

**Conclusion:**

The observer independent image capture of EFLCM allows quantitative measurements of fluorescence intensities and morphological parameters on a large number of cells. EFLCM therefore bridges the gap between the mainly illustrative fluorescence microscopy and purely quantitative flow cytometry. EFLCM can also be used as high content analysis (HCA) instrument for automated screening processes.

## Background

The introduction of laser scanning confocal fluorescence microscopy represented a major breakthrough in biology. The elimination of the disturbing out of focus light allowed the visualization of delicate sub-cellular structures that were hidden in the blur of thick samples [1-4]. Although the technique reveals structures with unprecedented sharpness, it also suffers from limitations. The images generate by scanning a sample with a single laser beam, where the in-focus signal passes a pinhole and is detected by a photomultiplier tube. This technique requires that the laser beam spends a few microseconds, depending on the signal to noise needed, on each and every point of the sample in order to produce a fluorescence signal. The size of the captured area is thus limited by the exposure time. To cover an area of 512 × 512 pixels the total exposure is in the range of 1000 ms, which is a relatively long time [5]. Capturing an area over one Megapixel is rarely practical. To produce a sufficient amount of photons strong lasers are used that cause significant bleaching on fixed samples or potential harm to living cells.

An alternative to single-beam scanning systems is the use of a Nipkow spinning disc for out of focus blur elimination. The spinning disc has a spiral array of twenty thousand pinholes, producing a multi-beam illumination and thus allowing an instant confocal signal capture of an entire field. The efficiency of the illumination is significantly enhanced by the use of a multi-microlens disk in front of the pinhole array disk (the Yokogawa head design), which focuses the laser beams onto the pinholes [6]. The major advantage of parallel beam illumination is that an image can be captured using cooled CCD cameras. Modern CCD cameras contain over a million individual photon-detectors with comparable quantum efficiency to photomultiplier tubes. The greatly increased speed and sensitivity of Nipkow spinning disc systems permits the use of low intensity excitation light resulting in reduced photo-damage [7,8]. Due to this advantage, spinning disc confocal microscopy is currently a preferred choice for live cell imaging [6,7,9].

CCD cameras can rapidly generate immense amount of image information. This, combined with the explosive increase in the computational power of modern desktop computers, make large-scale image capture, processing and analysis available for routine research use.

Due to the inherent human bias in the selection of an image area, fluorescence microscopy has for a long time been considered as mainly an illustrative tool. However, there is a great demand for quantitative analysis of fluorescence properties in cells [10,11] as illustrated by the extensive development of different flow cytometry techniques. Flow cytometry can objectively analyse hundreds of thousands of cells, but gives only limited and indirect (forward and side scatter) information about morphology. Already twenty-five years ago attempts were made to combine flow cytometry with a CCD-camera system producing still pictures showing preserved information of morphology and internal state of individual cells [12].

Automated, bias free image capture is the foundation of virtual microscopy. The general definition of virtual microscopy is the capture of a large amount of consecutive image tiles and the alignment of them into a continuous panorama, that is freely "zoomable" *in silico *[13-16]. Similar technology has for many years been used by astronomers to create panorama views of the night sky and by cartographers and military intelligence when examining series of satellite images of the earth. In pathology this is commonly achieved using line scanners with transmitted light illumination, where the linear-array detectors create a number of contiguous overlapping image stripes. During the last twenty years the development of several automated microscopy imaging techniques aimed the digitization of entire microscope slides and have led to the emergence of virtual histopathology [17].

An emerging field in biomedical imaging is the combination of fluorescence microscopy with automated capture techniques. Motorized XY-tables and high resolution cameras have been used to digitize entire slides using wide-field fluorescence illumination and 20X objectives [11]. Fluorescence microscopy and automated mosaic imaging repeated over time has been used to study avian embryo development. The embryo was imaged using a 10X/NA 0,25 objective and the multi-field XY-area typically covered 8 images (875 μm × 688 μm), at 10 different Z-focus planes [18]. In the field of automated identification and classification of cells it has been shown how to combine automated image capture with fluorescence microscopy for a high-throughput cell phenotype screening [19], [20]. Here one of the goals and achieved result was to capture one cell per image in a mutlti-array/microtiter plate fashion without the need to reconstitute a mosaic of all wells [20].

A commercial example is the MetaMorph system, using wide-field epifluorescence illumination, executing automated multi-field image capture and the creation of large mosaics [21,22]. Recently, original solutions with multi-objective arrays showed the possibility of fast wide-field capture over large areas [23]. In 1999, early attempts at virtual confocal microscopy were made by manually stitching confocal laser scanning microscopy images in Adobe Photoshop [24]. By the end of 2000, the power of automated Nipkow confocal microscopy and wide-field high-resolution cytometry was demonstrated in a new system. Due to the early version of the Nipkow module used, this system suffered drawbacks in illumination efficiency and was not sufficiently sensitive for low light fluorescence applications. Only 1–5% light throughput was reached, requiring long exposure times when using the confocal mode [25,26]. In conclusion, there are several examples of how fluorescence microscopy is combined with automated imaging, but not to the extent of high resolution confocal microscopy, sensitive for low light fluorescence applications for areas reaching the Gigapixel size. We wanted to combine the microlens enhanced Yokogawa Nipkow spinning disc (40% light throughput [7]) confocal microscopy technique with automated image capture and analysis in order to produce high resolution images covering a very large area of interest, which could be analyzed automatically. Here we present the Extended Field Laser Confocal Microscopy (EFLCM) – method, where the end result is a Gigapixel mosaic showing confocal fluorescence information from several thousands of images at the maximal resolution achievable by a light microscope.

## Methods

### Hardware

In this study we have used a custom built instrument based on the UltraVIEW LCI system (Perkin Elmer, USA). The system includes: A motorized Axiovert fluorescence microscope 200 M (Zeiss GmBH, Göttingen, Germany), a CSU10 Yokogawa head – a multilens/multi pinhole array aperture (Yokogawa, Japan), a motorized XY-table (Märzhauser, Germany), an ORCA ER cold CCD camera; detector array 1344 × 1024 px (Hammamatsu, Japan), a 3-line Argon-Krypton Laser; 488 nm, 568 nm, 647 nm (Melles Griot, USA) and a mercury lamp (Osram) for 365 nm illumination. The following objectives were used in this study (Zeiss):

10X/NA 0,25 Ph1 ∞/- A-Plan,

16X/NA 0,5 Imm Plan – Neofluar ∞/0,17

63X/NA 1,25 Oil Ph3 Plan – Neofluar ∞/0,17.

### Software

All hardware was controlled and automated using programs developed by us (see the Results section), in the programming environment OpenLab Automator (Improvision, Perkin Elmer). The raw multi-field image stacks were processed in and mosaics assembled using the ImageLab/Virtual Microscope software (Aragon System, Östersund, Sweden). The programs are available at the following web sites [27] and [28].

### Preparation of biological samples

Both paraffin embedded and frozen slides of rodent tissues were labeled either by immunofluorescence or conventional histological (HE) staining. In vitro cultured cells were grown on cover glass in monolayers, alternatively suspension cells were centrifuged on glass slides using cytospin centrifuge (50.000/100 μl, centrifuged at 1500 rpm for 4 minutes). Immunofluorescence staining of viral antigens and transfection of monolayer cells with plasmids carrying GFP fusion proteins were carried out as described in earlier publication [29]. The cells were fixed using methanol:aceton 1:1 mixture or 1% formaldehyde in PBS.

All cells used in this study were cultured in IMDM (Sigma) supplemented with 10% FCS (Sigma) and 50 μg/ml Gentamicin (Sigma). Cell suspensions were grown in a humidified incubator at 37°C in an atmosphere containing 5% CO_2_.

## Results

### Automating the image capture process – the QuantCapture program

The multi-beam illumination of the microlens enhanced Nipkow spinning disc, the Megapixel image capture of cold CCD cameras combined with the possibility to automate image capture, inspired us to explore the possibility of generating confocal images over large areas. To achieve this we have assembled a fully motorized system based on an inverted fluorescence microscope equipped with a motorized XY-table. We have opted to control all motorized units of the microscope; illumination shutters, excitation/emission filter wheels and the different capture modes of the cold CCD camera. We control these units via QuantCapture4, a customized program that we have developed using OpenLab Automator. The OpenLab Automator is a high level, symbol based, visual programming language. It contains "action icons" for every aspect of hardware control, image handling, measurement functions, standard logical and arithmetic operations, as well as memory and file handling tools. These icons can be combined into complex networks, resembling flow charts, individually designed to execute intricate sequences of actions automatically. The Automator allows the declaration of numeric and string variables and variable arrays. It also allows the creation of subroutines that can exchange variables with the main program. The main program and the subroutines can then be linked into multi-task networks.

**Figure 1:**
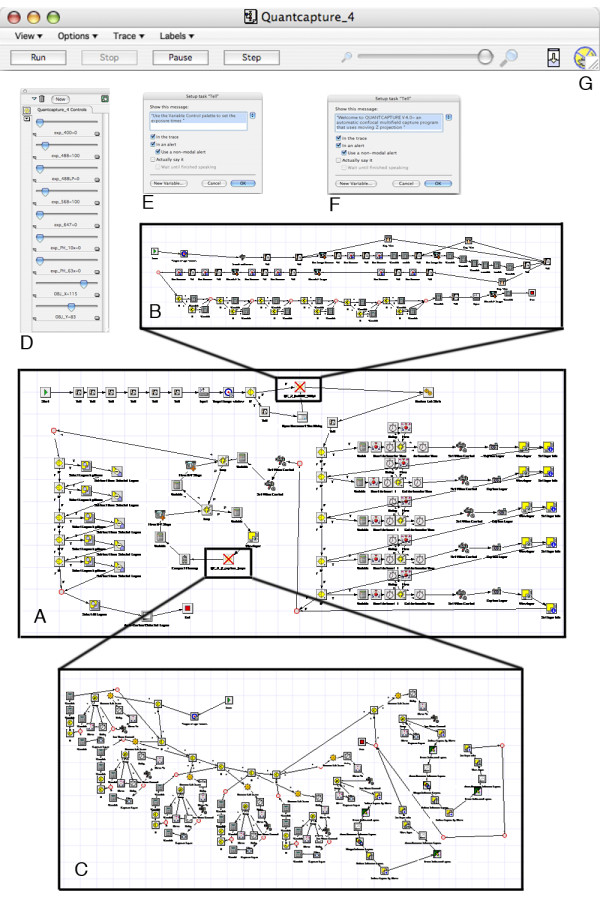
A-G. A-C Flowcharts of QuantCapture4, the program controlling the automated image capture, developed by us in the visual programming environment OpenLab Automator. A: Main program. B: Subroutine 1, Find Four Corners. C: Subroutine 2, Z Capture Loops. D: Slider control interface: Settings for excitation wavelengths, exposure times and field of view of the objective (XY-dimensions). E: Pop-up message, to guide the user through the settings of parameters. F: Pop-up Welcome message. G: Head interface of QuantCapture4, shows program status and control panel for start (Run), pause and ending the capture (Stop).

In order to automatically capture confocal images over large areas and multiple focal planes we have developed the program QuantCapture4. The QuantCapture4 design includes a main program (Fig. 1A,F) and two subroutines (Fig. 1B,C). When starting the program the user defines excitation wavelengths, exposure times and the field of view of the objective (XY-dimensions) using a slider control interface (Fig. 1D). To define the extent of the capture area the main program invokes the first subroutine, called "Find Four Corners" (Fig. 1B). Here an area of interest can either be manually selected by looking into the microscope and choosing the relevant field or can be numerically defined as fixed coordinates. The number of images needed to cover the selected area is calculated and sets the values for how many rows and columns of tiles that will be captured. To allow for further image processing and pixel precise alignment of the mosaic tiles, overlapping areas can be defined by appropriately selecting the X and Y objective dimensions using the slider control interface (Fig. 1D).

### Moving Z capture

Confocal microscopy produces optical sections of focal planes that are free from out of focus blur. Inherently, however, it also restricts the collected information to the actual selected focal plane. To overcome this, microscopists regularly collect images from a series of individually captured optical sections at different Z-axis positions. These images are then examined individually or projected into a new summarized image using different projection algorithms (maximum intensity, sum, average, depth shading, surface shading etc [30]). High-resolution objectives (NA > 1) regularly mean high magnification and small field of view. Therefore, the capture of thousands of consecutive tiles is required to cover large areas at high resolution. Due to the fact that neither microscope slides nor mounted objects are perfect horizontal and no Z-motor work without a Z-drift, a capture over a large area often means that the sample is present in wide range of different focal planes. Generally different methods of auto-focusing are employed to keep the capture in focus over large areas. These algorithms are always relatively time consuming and also expose the sample for potential photo bleaching, without gaining information. To ensure that the sample stays in focus and to assure that all visual information of the sample is preserved, we have developed a capture method that we call the "Moving Z Capture", invoked in the second subroutine "Z Capture Loops" (Fig. 1C). Here we let the Z-motor move in pre-set steps from top to bottom focus along the Z-axis, while the cold CCD-camera collects all signals during one total exposure, without closing the shutter in between the Z-steps. Using this method, only one image consisting of all signals through the entire Z-axis is produced. The Nipkow spinning disc assures that only confocal signals from the different focal planes can reach the camera. When defining the capture area in the subroutine "Find Four Corners" (Fig. 1B), the program also prompts the user to define the top and bottom focus planes for the four corners of interest. The program calculates the absolute minimum and maximum Z-positions, shows the total focus range and prompts the user to decide how thick theoretical optical sections should be. The focus range divided by Z-step thickness gives the number of steps the Z-motor will move through and delay at with one given exposure time. The number of Z-steps will affect the total exposure time. The technique requires the exposition time of the camera to be precisely synchronized with the Z-motor travel time. We solved this challenge of synchronization by including a dummy capture cycle at every wavelength, which calculates the sum of the exposure times at every step together with the corresponding travel time of the Z-motor. This value sets the total exposure time for each wavelength in the "Z Capture Loops", the subroutine controlling the interactions of the Z-motor, emission and excitation filter wheels with the CCD camera. To correct for camera background noise, the program carries out automatic image subtractions using a "dark background" image. This "dark background" image is the noise the camera detects with closed illumination shutters, during one total exposure time.

In a Moving Z capture, the camera acts as an integrator, summing the image intensity at each pixel through the Z-depth travel. This produces a true sum projection of all signals present in the Z-depth/focus range. We found that the quality of a Moving Z image is better (higher S/N ratio) when compared to an ordinary sum-projection of individually captured Z-layers at identical Z-depth (see the figure MovingZcomparison, in Additional file 1). The exposure time at each step through the Z-stack is identical between the two types of capture techniques. The difference is that in the Moving Z capture the camera also collects signals even in between the steps, making the exposure time through the part of the Z-depth that actually displays the sample relatively longer compared to a single layer capture. Moreover, a sum-projection of individually captured Z-layers multiplies the background camera noise by the number of Z-layers. As we have measured, the background cold CCD camera noise contributes only once to the final image generated by the Moving Z capture. This background is also removed using the total exposure "dark background" image. The Moving Z technique has proved very appropriate for the purpose of the EFCLM method; to build mosaics in the XY-dimension with preserved Z-dimension information but without having to save individual Z-planes.

The program also allows periodic saving of the captured images, meaning that hard disc storage space is the only limitation of the captured area.

A modified version of QuantCapture4 has been developed to capture a full Z-depth image, at different excitation wavelengths from each well, in a 384-well plate.

### ImageLab/Virtual Microscope

**Figure 2:**
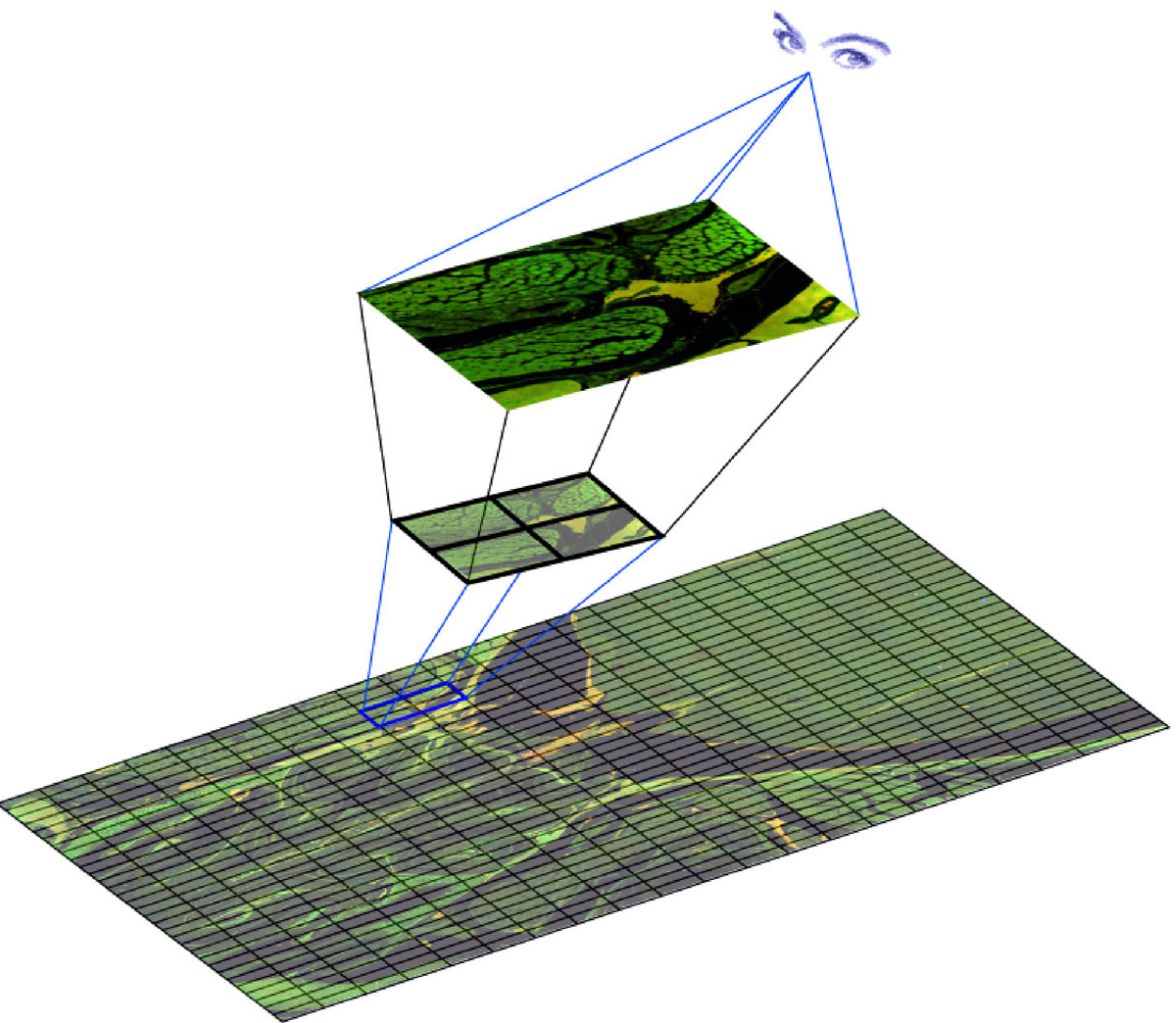
This schematic image illustrates the virtual microscopy features of a montage presented in the ImageLab/Virtual Microscope software. Allow real-time browsing by building a representation of images from the aligned stack "on the fly".

**Figure 3:**
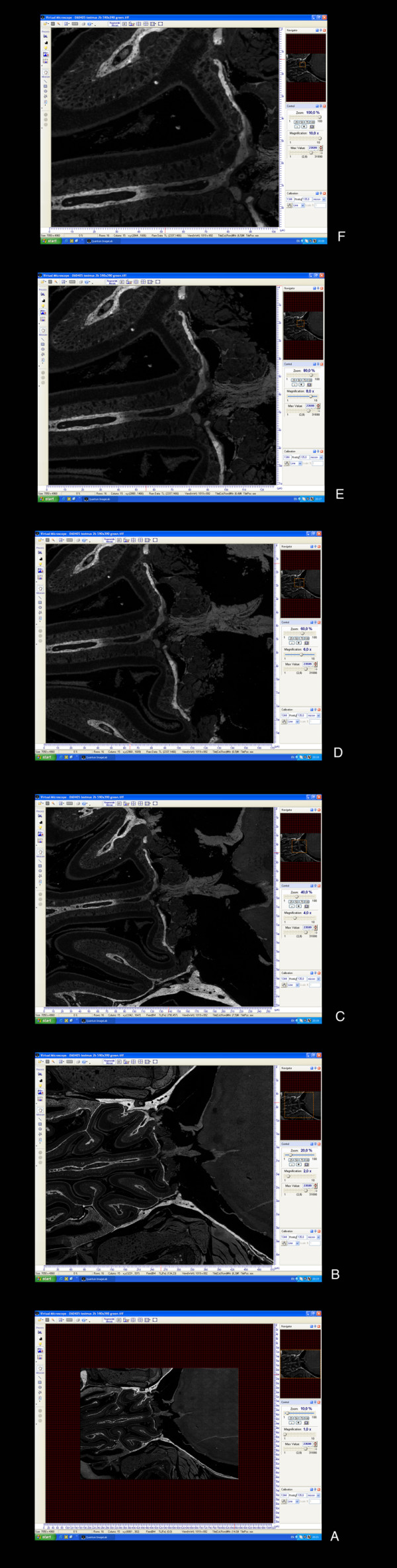
Interface of the ImageLab/Virtual Microscope software: Real-time browsing, thumbnail window for navigation and zoom in application.

To handle hundreds, or even thousands, of Megapixel images is a formidable computational challenge for desktop computers. Here we show that the recently developed ImageLab/Virtual Microscope software (Aragon System, Sweden) offers a viable solution to deal with this amount of image information. The ImageLab/Virtual Microscope software performs satisfactorily on a PC with 1 GB working memory and 2 GHz processor, using the Windows operating system. The Virtual Microscope image montage application uses the raw image stack, created in QuantCapture4, to construct a perfectly aligned mosaic of tiles seamlessly joined together. The assembled mosaic can be viewed at different magnifications, with free movements in any direction and with the possibility to zoom-in on any details, just like a real microscope (Fig. 2, 3).

The only requisite input value is the dimension of the captured area, as defined by rows and columns in the QuantCapture4 program. To address optical and mechanical imperfections inherent in an automated microscope system [15,31], several image processing operations are used in the construction of the final, aligned mosaic. Potential imperfections corrected by the Virtual Microscope program are:

#### Imperfect XY-motor mechanics

**Figure 4:**
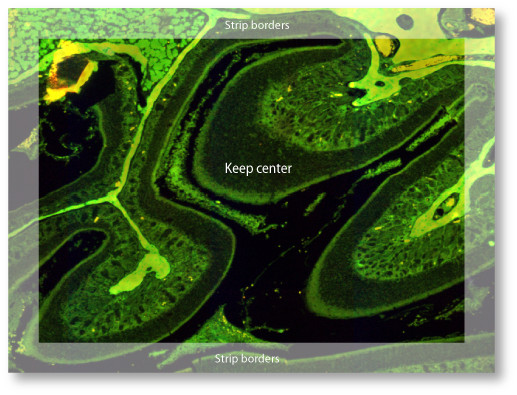
To aid image processing for pixel precise alignment: Images are captured with overlapping borders.

The precision of the XY-stepping motor is in the range of 1 μm. Consequently, additional computational solutions are required to create pixel precise matching. First, images are captured with an optimal overlap and are therefore cropped (Fig. 4) before pixel precise matching. For example, when using a 63X objective, all images are captured using a 20 μm overlap. In our case we found that edge detection paired with Fourier space correlation is the best method of finding precisely overlapping pixel patterns [32]. We also use noise reduction to further facilitate alignment [33,34].

#### Uneven illumination

**Figure 5:**
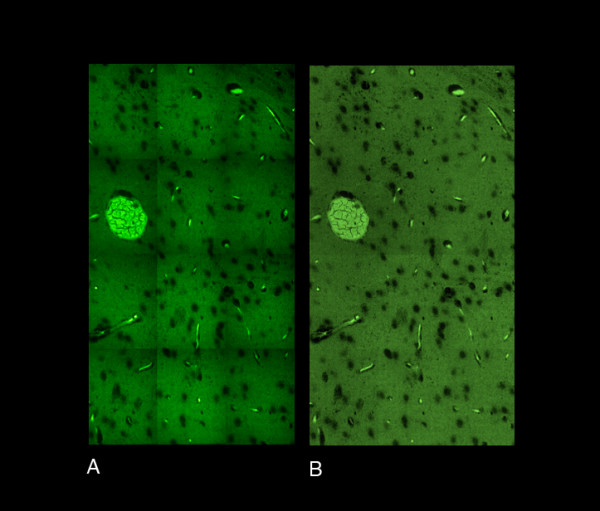
Image processing in ImageLab/Virtual Microscope software: Flat-fielding, to compensate for uneven illumination artifacts. A: Vignetting artifacts before Flat-fielding. B: After Flat-fielding.

Factors related to the design of the light path between the camera and the microscope or from uneven geometry of the laser beam may lead to illumination inhomogeneities [35]. They are barely detectable on individual images, but their periodic nature can create disturbing vignetting artifacts on a multi-field assembled mosaic. (Fig. 5A). We carry out flat-fielding (Fig. 5B) by multiplying each raw image with a template. This template can either be user supplied or calculated by statistical methods.

#### Misaligned camera

Even a slight misalignment of the camera in relation to the position of the stage can generate significant difficulties when large arrays of images are captured. If the camera is not perfectly parallel to the stage a drift will accumulate and make it impossible to create an aligned mosaic. Rotating all the images prior to alignment can compensate for this defect. We found the angle of rotation by taking the median of all shifts necessary to align each image with its left neighbor. This means that in order to achieve perfect alignment in the stack it is necessary to first rotate and then register the images. The flat-fielding operation is also essential to enable alignment.

**Figure 6:**
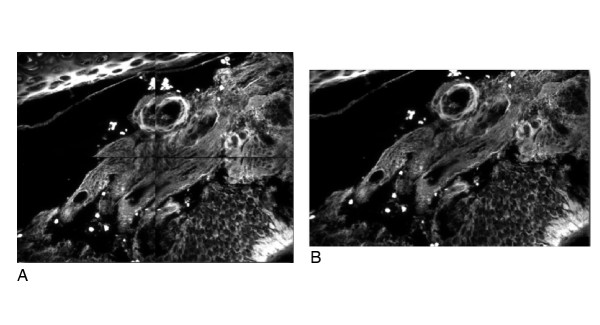
**Image processing in ImageLab/Virtual Microscope**: A: Montage of four images before cropping overlapping borders, pixel precise alignment and flat-fielding. B: The final montage after corrections (after cropping, pixel precise alignment and flat-fielding).

After corrections the result is a new image stack where the images are of even brightness and in perfect alignment with each other (Fig. 6A,B).

### Visualization using in silico image browsing

We could demonstrate that the visualization features of theImageLab/Virtual Microscope software allow real-time browsing by building a presentation from the aligned stack "on the fly" (Fig. 2). Even with the high performance of modern desktop computers, a brute force implementation of stack visualization is not practical. To align and combine all image tiles into one huge, "browsable" mosaic would generate an image so big that an ordinary operating system would not be able to load it. The performance demands of the scaling operations involved in presenting the user view would also be prohibitive. As an example, a capture of 40 × 30 images with an individual size of 1200 × 1000 pixels would generate an image approximately 1.2 Gigapixels large (after cropping overlapping borders). If each pixel is captured in 3 color planes at a 12-bit resolution each – requiring 2 bytes of storage – the resulting image would be roughly 8 Gigabytes in size. To create a user-view we instead used a layered approach that both scales down the memory requirements of the OS and the performance demand on the application. The Virtual Microscope visualization application constructs the user-view by assembling image data from those tiles in the actual view. This mode is used whenever the magnification is high (Fig. 3B,F). At low magnification this strategy would be unnecessarily slow because a lot of tiles would have to be referenced to construct the view. Instead the view is then constructed from a moderately sized montage with maximum width/height of 5000 pixels. No observable details are lost using this approach because even the intermediate montage contains more details than a computer screen can present.

### Image analysis and quantitation

**Figure 7:**
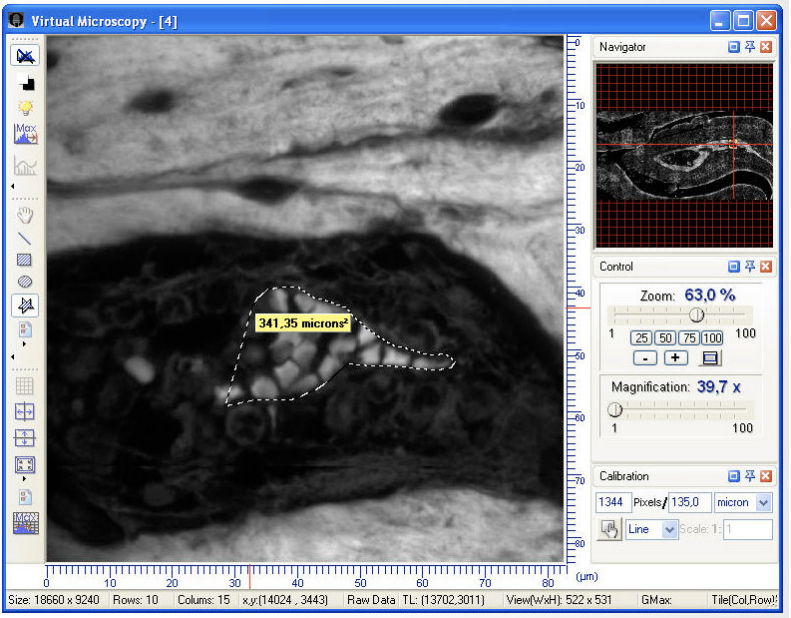
**Tools for image analysis in ImageLab/Virtual Microscope**: To aid the interpretation of biological data the software allows geometrical measurements for distance and area.

To aid the interpretation of biological data, the final mosaic can be further enhanced using various tools within the ImageLab/Virtual Microscope software: Contrast stretching, histogram equalization, morphological operations (for object identification and feature extraction) and geometrical measurements, for distance and area (Fig. 7). Moreover wavelet-shrinkage on the view area can be applied for noise removal.

### Breaking the Gigapixel limit of a confluent confocal image

**Figure 8:**
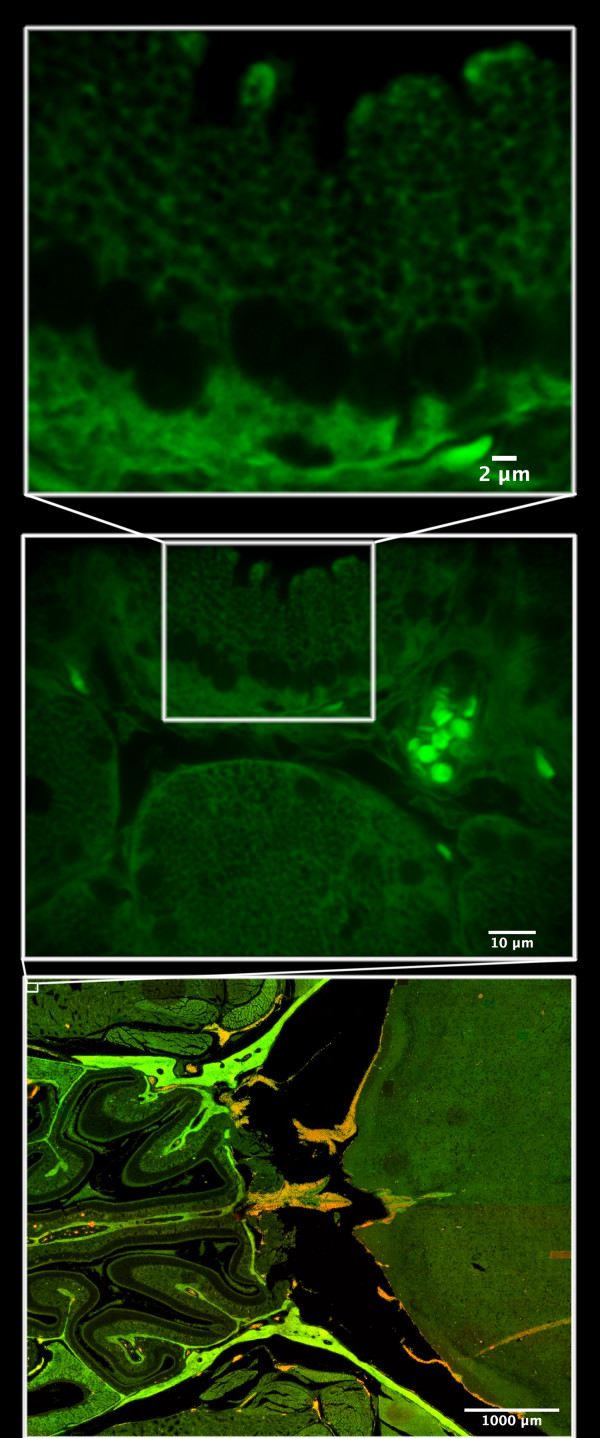
**Breaking the Gigapixel limit of a confluent confocal panorama image**: A 2,8 Gigapixel size confocal mosaic, built up from 3000 (50 columns × 60 rows) individual confocal images (A), using the ImageLab/Virtual Microscope software. The internal structures of a mouse head are visualized with eosin fluorescence at 63X/NA1.25, individual images cover an area of 135 μm × 103 μm (B), captured at binning 1 (1344 × 1024 pixels) and 200 pixels overlap. After image processing in the ImageLab/Virtual Microscope software the final seamless confocal panorama show an area of 5,75 mm × 4,98 mm. The original resolution of each individual image is preserved in the final montage, allowing the user to zoom in on details at single cell level (C).

To demonstrate the power of automated parallel beam confocal microscopy, driven by the QuantCapture4 program, combined with the high performance of image stack correction and construction of seamless panoramas, performed by the ImageLab/Virtual Microscope, we have created a 2,8 Giga pixel continuous confocal mosaic (Fig. 8A). This extended field image, built up from 3000 individual confocal images (50 columns × 60 rows), shows the internal details of a mouse head visualized with eosin fluorescence at a high optical resolution (63X/NA 1,25). The captured area was 5,75 × 4,98 mm and each image field cover 135 μm × 103 μm, at 10 pixel/μm resolution. Using the QuantCapture's "Moving Z" technique each of the 3000 images summarize confocal information from a 5 μm thick tissue section. To assure that the sample stayed in focus over the large field of interest the moving Z capture used a 39 μm Z-travel distance, divided in 20 steps, with a 100 ms delay time at every step. The total exposure time for a Moving Z capture, at one wavelength, was approximately 10 s (400 images/hour). To allow pixel precise matching of structures in the final mosaic, individual images (1344 × 1024 pixels in size) were captured with 200 pixels overlap (resulting in 1144 × 824 pixels in size). The final mosaic display 57 200 × 49 440 pixels = 2 827 968 000 pixels or 2,8 Giga pixels, free to browse and to zoom in on for maximal resolution (Fig. 8B–C). All individual images were flat-fielded and corrected for 0.5 degrees of stack rotation. The intermediate montage used for low magnification virtual browsing is 10% of the full size. A higher resolution image can be found at .

### Examples of possible applications

**Figure 9:**
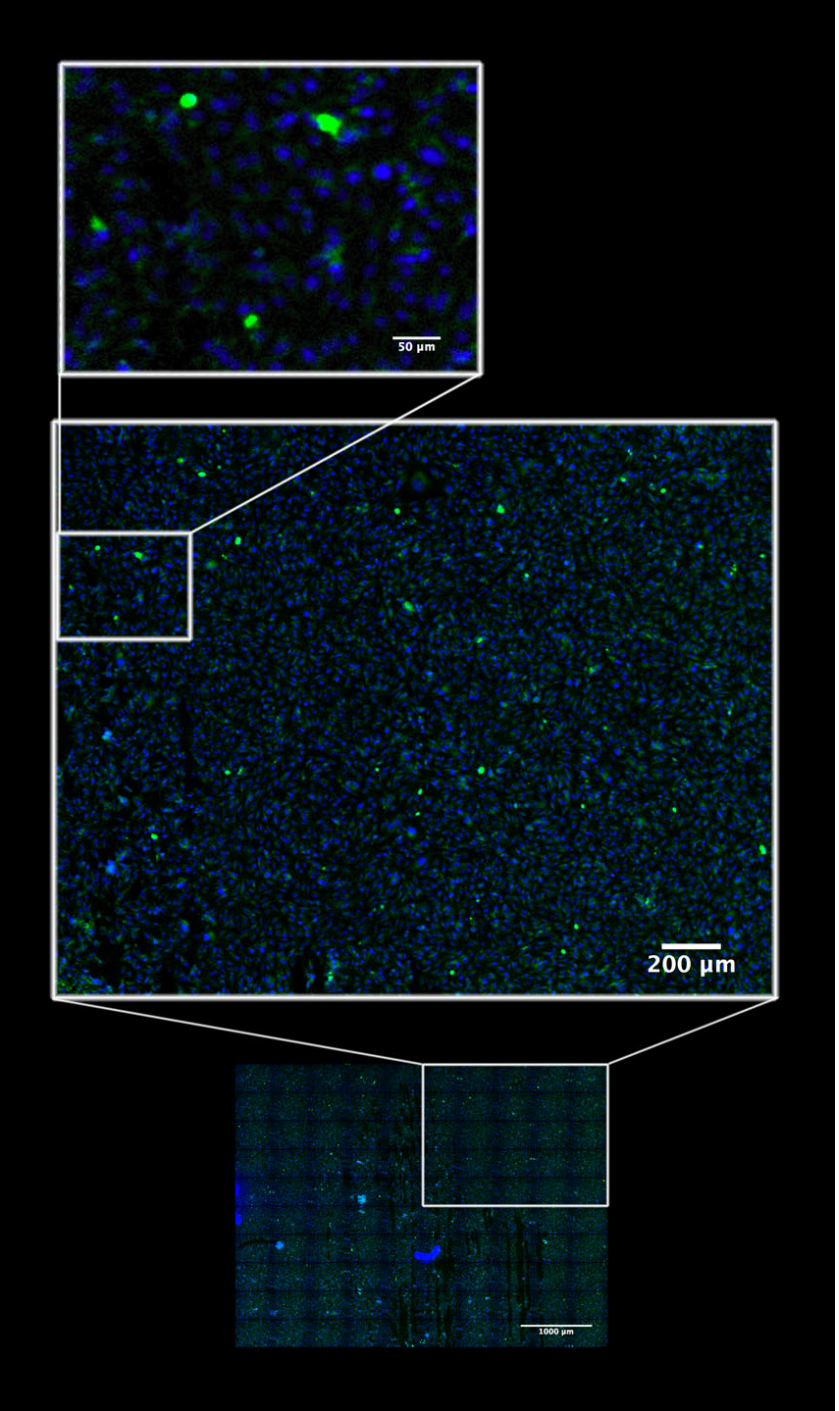
**Example of application of the EFLCM method**: To visualize thousands of cells growing in monolayer – for automatic and objective quantitation of epigenetic effects of the transformation associated viral nuclear antigen HHV8 LANA fused to green fluorescence protein in a transient transfection assay. The blue color is Hoecsht 33342 fluorescence showing DNA. The mosaic is built up from 100 images (10 columns × 10 rows) captured at 16X/NA O.5. Individual images covers an area of 522 μm × 398 μm. Images are captured at binning 1 (1344 × 1024 pixels) with 100 pixels overlap resulting in a final area of the seamless panorama, after image processing in the ImageLab/Virtual Microscope, of 4,82 mm × 3,58 mm.

**Figure 10:**
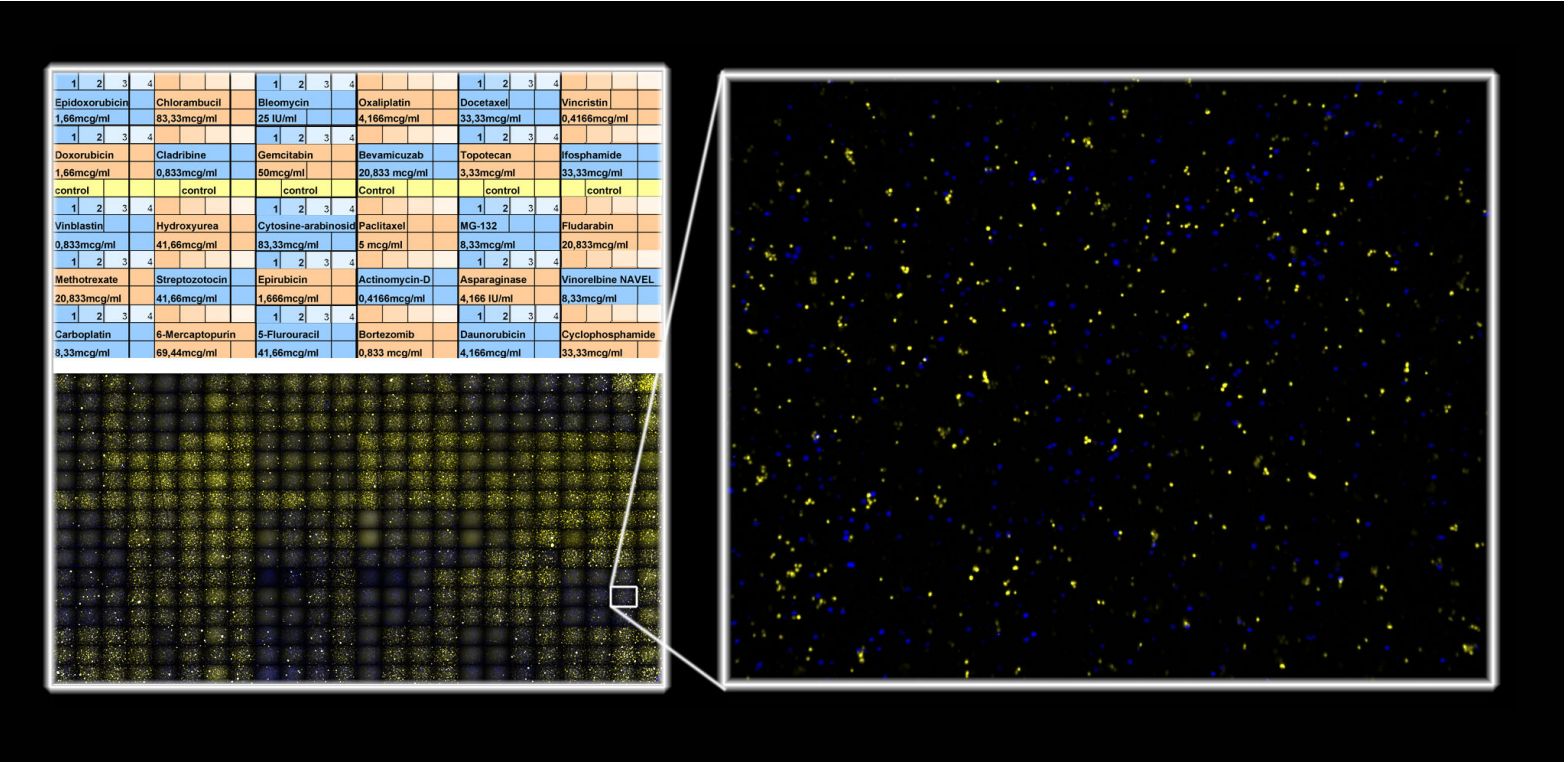
**Example of an application of the EFLCM method**: To screen the effect of 30 different anti-cancer drugs, preprinted on a 384-well plate and incubated for three days with primary tumor cells from one patient. Using live/dead fluorescence cell discrimination to determine a drug sensitivity profile for primary human tumor cells with the aim of assisting individualized assay guided cancer therapy.

We have used the described method in a variety of diverse applications. Such as to visualize entire mouse embryos in gene knockout experiments [36], in quantitation of epigenetic effects of transformation associated viral nuclear antigens fused to green fluorescence protein in transient transfection assays [29] (Fig. 9), in finding virus infected cells with a rare type of latency program on immunofluorescent labeled suspension cells on cytospin prepared slides, when determining the anti-cancer drug sensitivity of primary human tumor cells on 384 well plates (Fig. 10) [37,38] or measuring the killing effectiveness of human NK and CTL cells against fluorescently labeled target cells in 384-well plate assays [39].

## Discussion

The introduction of Nipkow spinning disc confocal technology, the ability to automate the actions of a motorized microscope and the increasing power of desk top computers have stretched the boundaries of microscopy and image acquisition. Combining these factors with advanced *in silico *image processing; we have shown that it is possible to create high-resolution confocal panoramas of specimens covering entire slides. Here we presented the Extended Field Laser Confocal Microscopy (EFLCM) method, allowing the presentation of a Gigapixel mosaic that combines information from several thousands of images showing both X,Y and Z-information.

In our view one of the most important emerging trends in microscopy is the automation of advanced microscopy techniques. Confocal microscopy is mainly used today as an illustrative tool. There is a need to use confocal microscopy as a technique for objective measurements. Automated, observer independent image capture can eliminate the greatest source of error in quantitative microscopy – the bias of the subjective field selection. It also produces images captured under identical conditions, preventing inhomogeneous exposure and differences in bleaching of the samples and thus allowing a more reliable quantitation of intensity analysis.

We have used the OpenLab Automator, a high level visual programming language, to create programs that drive automated microscope functions. Previously we have shown that hardware controlling programs created in a similar programming environment, ISee (ISee Imaging Systems, Raleigh), can be very useful in rapidly generating deconvolved 3D images [40]. Through this publication we would like to advocate that high level visual programming languages enable users to tailor imaging applications, without extensive knowledge of complex computer languages.

To perfectly assemble very large arrays of images, with pixel precise matching, is however beyond the capacity of the OpenLab programming environment. To meet this challenge, we have developed the ImageLab/Virtual Microscope software. In this software the image stack is processed for imperfections and a new stack of perfectly aligned and intensity corrected images are constructed to create a seamless panorama. After this digitalization process the actual browsing of the specimen is independent of the physical microscope. Virtual microscopy has rapidly gained popularity in clinical pathology and medical teaching where bright field illumination allows the use of line scanners, permitting very rapid image acquisition in the Gigapixel range [16,17,41-45]. Although EFLCM uses a process of image capture (approximately 400 images/hour using a 63X objective, the time is dependent on the thickness of the z-depth, which increases with size of field) that is considerably slower than line scanners, its ability to detect very weak fluorescent signals, at different wavelengths and at the maximum resolution of a light microscope, gives it a great advantage. Moreover, the "Moving Z" confocal illumination technique generates images that quantitatively preserve all visual information from different focal planes thus allowing precise measurements within the context of preserved biological structures.

**Figure 11:**
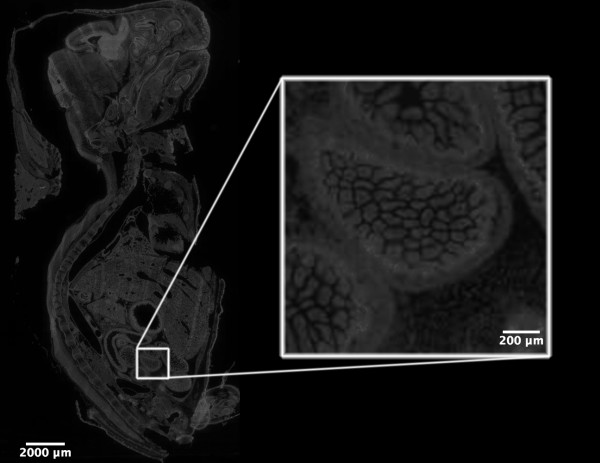
**Example of a semi-confocal application of the EFLCM method**: Using EFLCM we easily capture images of an entire slide, here showing Hoecsht 33342 fluorescence from DNA in all cells of a whole mouse fetus. The mosaic is built up from 782 images (17 columns × 46 rows) captured at 10X/NA O,25. Individual images covers an area of 835 μm × 636 μm. Images are captured at binning 2 (672 × 512 pixels) with 100 pixels overlap resulting in a final area, after image processing in the ImageLab/Virtual Microscope, of 12,1 mm × 23,6 mm.

The extensive use of transgenic and gene knockout technology rapidly generates genetically modified organisms, often with very complex and unpredictable phenotypes. The possibility to automate confocal microscopy imaging of entire mouse embryos [36] and fetuses (Fig. 11), zebrafishes, nematodes and fruit flies, all at maximal resolution, may become a very valuable tool for functional genomic research. EFLCM will allow the measurement of the expression levels of mRNAs of individual genes by fluorescent in situ hybridization, the levels of individual proteins by immunofluorescent staining or the activity of selected promoters after targeted insertion of GFP expression cassettes in tissue sections. Using EFLCM in multi-wavelengths mode will permit the analysis of biochemical effects of selected genes on tens of thousands cultured cells after introduction by transient transfection.

Adopting EFLCM to analyze 384-well plates can provide a simple instrumental solution for academic researchers helping to adopt high content analysis techniques in their everyday work. It may create a technological platform for large-scale analysis of living tumor or immune effector cells in routine clinical laboratory praxis to assist individualized assay guided therapy, as we have recently shown [37-39].

## Conclusion

We have developed a technique that we call Extended Field Laser Confocal Microscopy (EFLCM). It is based on automated confocal microscopy and high performance image processing *in silico*. We have shown that it is possible to produce seamless confocal panoramas, using an image capture technique developed by us in the visual programming language Openlab Automator followed by advanced image processing and mosaic assembly in the ImageLab/Virtual Microscope software. We have shown that EFLCM is a useful tool for qualitative and quantitative analysis of histological slides, embryos, fetuses, a large number of transfected cells and of samples in 384-well plates.

## Competing interests

EF and LS declare that they have no competing interest. PS and CS are owners of Aragon system that has developed the ImageLab/Virtual Microscope software.

## Authors' contributions

EF and LS conceived the study and carried out the programming for automated image capture. PS and CS developed the ImageLab/Virtual Microscope software.

## Pre-publication history

The pre-publication history for this paper can be accessed here:



## Supplementary Material

Additional file 1 A. Moving Z capture: An image captured through a Z-depth of 39 μm during one total exposure. The travel through the 39 μm is divided into 20 steps, with a 100 ms delay time at every step, without closing illumination shutter in between steps (it takes approximately 10 s to perform this Moving Z capture, at one wavelength -> 400 images/hour). B. Layer 9 of a 20 layer-stack of individually captured Z-layers. Captured through the identical Z-depth as A, 100 ms exposure at every layer. C. Sum-projection of all 20 layers in the stack of individually captured Z-layers. Captured through the identical Z-depth as A, 100 ms exposure at every layer (it takes approximately 14 s to perform individually captured images through Z, at one wavelength -> 257 images/hour). D. Maximum Intensity-projection of all 20 layers in the stack of individually captured Z-layers. Captured through the identical Z-depth as A, 100 ms exposure at every layer (it takes approximately 14 s to perform individually captured images through Z, at one wavelength -> 257 images/hour). Click here for file
