# Supplementary figures and images for: Extended Field Laser Confocal Microscopy (EFLCM): Combining automated Gigapixel image capture with in silico virtual microscopy
Source: BMC Med Imaging. 2008 Jul 16;8:13. doi: 10.1186/1471-2342-8-13 (PMC2515298; doi:10.1186/1471-2342-8-13)

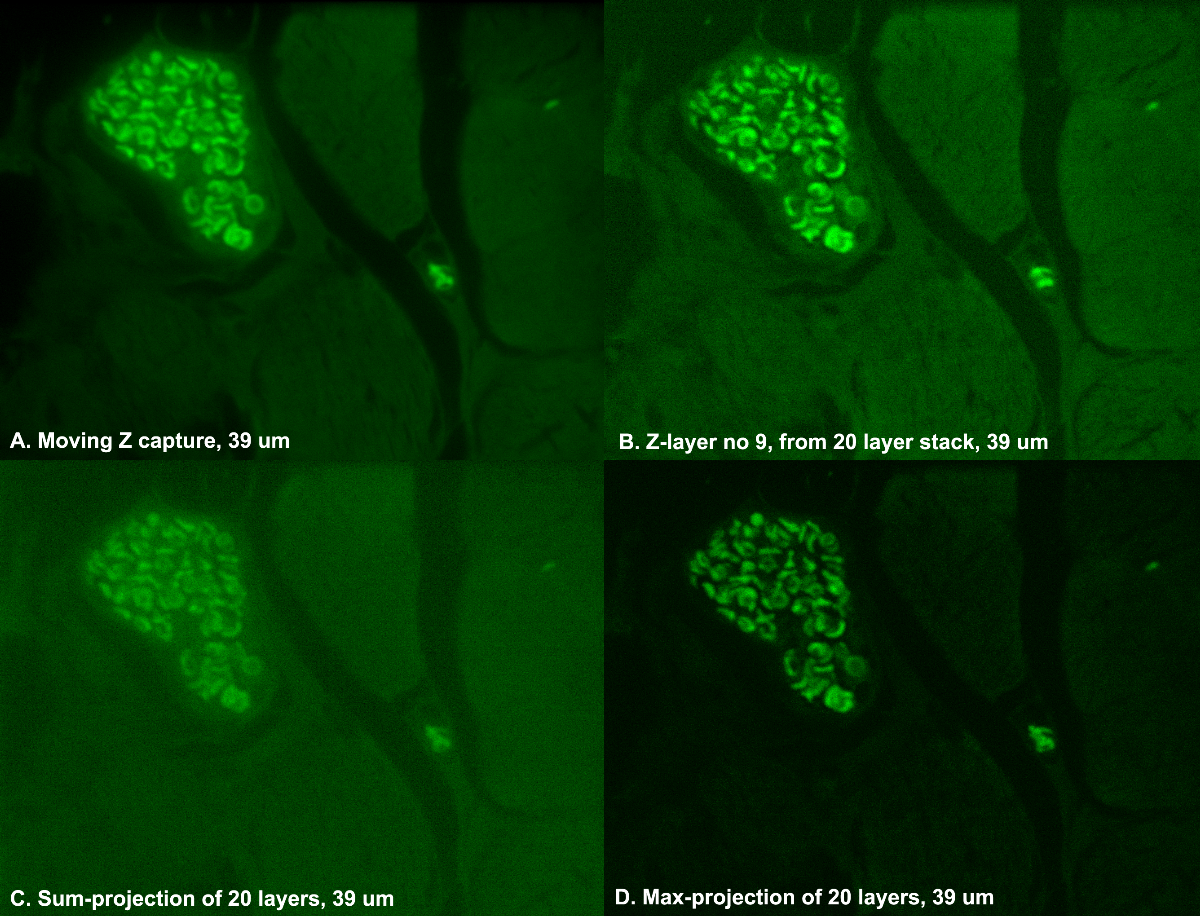

Supplement: Additional file 1 — A. Moving Z capture: An image captured through a Z-depth of 39 μm during one total exposure. The travel through the 39 μm is divided into 20 steps, with a 100 ms delay time at every step, without closing illumination shutter in between steps (it takes approximately 10 s to perform this Moving Z capture, at one wavelength -> 400 images/hour). B. Layer 9 of a 20 layer-stack of individually captured Z-layers. Captured through the identical Z-depth as A, 100 ms exposure at every layer. C. Sum-projection of all 20 layers in the stack of individually captured Z-layers. Captured through the identical Z-depth as A, 100 ms exposure at every layer (it takes approximately 14 s to perform individually captured images through Z, at one wavelength -> 257 images/hour). D. Maximum Intensity-projection of all 20 layers in the stack of individually captured Z-layers. Captured through the identical Z-depth as A, 100 ms exposure at every layer (it takes approximately 14 s to perform individually captured images through Z, at one wavelength -> 257 images/hour). [file 1471-2342-8-13-S1.tiff]
